# Cerebral Radionecrosis Following Stereotactic Irradiation of Skull Bone Metastases: A Diagnostic Pitfall to Be Aware of

**DOI:** 10.3390/diagnostics16111628

**Published:** 2026-05-26

**Authors:** Gianluca Ferini, Anna Viola, Valentina Zagardo, Antonio Pontoriero, Saveria Spadola, Gianluca Scalia, Giuseppe Emmanuele Umana

**Affiliations:** 1 REM Radioterapia srl, 95029 Catania, Italy; 2 Department of Medicine and Surgery, University Kore of Enna, 94100 Enna, Italy; 3 Radiation Oncology Unit, Fondazione Istituto Oncologico del Mediterraneo, 95029 Catania, Italy; 4 Department of Medicine and Surgery, University of Messina, 98125 Messina, Italy; 5 Pathology Unit, Cannizzaro Hospital, 95126 Catania, Italy; 6 Unit of Neurosurgery, Department of Head and Neck Surgery, Garibaldi Hospital, 95124 Catania, Italy; 7 Department of Neurosurgery, Trauma and Gamma Knife Center, Cannizzaro Hospital, 95126 Catania, Italy

**Keywords:** skull bone metastasis, stereotactic radiosurgery, brain, radionecrosis, breast cancer

## Abstract

We report an unusual case of brain radionecrosis following fractionated stereotactic radiotherapy (FSRT) delivered to a calvarial metastasis in a patient with metastatic luminal A breast cancer. A 40-year-old woman developed three subcentimetric brain lesions during follow-up, initially interpreted as new metastases and treated with FSRT. Subsequent radiological progression and advanced imaging suggested radionecrosis, which was confirmed histologically after surgical resection. Retrospective dosimetric analysis revealed that all lesions arose within the high-dose isodose region of the previously irradiated skull metastasis, despite compliance with established brain dose constraints. This case highlights a previously unreported risk of brain radionecrosis after stereotactic irradiation of skull bone metastases and underscores the need for caution in treatment planning and imaging interpretation.

A 40-year-old woman with a history of right-sided locally advanced luminal A breast cancer, treated in 2020 with neoadjuvant chemotherapy followed by surgery, adjuvant endocrine therapy, and radiotherapy, developed diffuse skeletal disease two years later. Imaging revealed multiple lytic bone metastases, including a large lesion extensively eroding the right parietal calvarial bone and causing a mild impression on the underlying brain parenchyma (yellow arrow in Figure 1A).

This finding prompted the simultaneous initiation of systemic therapy with ribociclib and radiotherapy to all symptomatic bone metastases, with careful site-by-site assessment of the safety of a concomitant approach [1]. Antiresorptive therapy with denosumab was also started. Given the need for dose escalation beyond a purely palliative intent for the calvarial lesion, fractionated stereotactic radiotherapy (FSRT) was selected. The skull bone metastasis was treated in May 2022 with a total dose of 30 Gy delivered in five consecutive daily fractions of 6 Gy.

Treatment was delivered using a TrueBeam Novalis STx linear accelerator (Varian Medical Systems, Palo Alto, CA, USA) with five non-coplanar volumetric arcs, employing couch angles of 30°, 60°, 270°, 300°, and 330°. This configuration was considered the optimal compromise in terms of dose homogeneity, conformity, and sparing of adjacent organs at risk (OARs) [2]. The resulting target dosimetric quality was acceptable, with a maximum dose (D_max) of 101.2%, and a V_98% of 99.5%, indicating high dose homogeneity, and a Paddick conformity index of 0.74. The relatively suboptimal dose conformity was likely related to the shell-like concave–convex geometry of the calvarial target, limiting further optimization. With respect to the brain, the volume receiving at least 24 Gy (V_24Gy) was limited to 19.11 cc, V_28.8Gy was 11.79 cc, and V_30Gy was 0.09 cc, while all other OARs were well within accepted dose–volume constraints. Although the dosimetric evaluation primarily reflects multifractionated stereotactic regimens, we additionally report V_12Gy and V_18Gy, which are more commonly described in the single-fraction stereotactic radiosurgery literature, together with mean brain dose for completeness and in order to improve comparability with published radionecrosis risk data. V_12Gy and V_18Gy were 249.72 cc and 147.62 cc, respectively, while the mean brain dose was 3.37 Gy [3,4].

A subsequent magnetic resonance imaging (MRI) follow-up demonstrated persistent stability of the treated calvarial lesion at all time points. Brain MRI remained negative until July 2025, 38 months after radiotherapy to the calvarial metastasis, when three distinct subcentimetric contrast-enhancing lesions appeared within the brain parenchyma. These lesions were separated from each other by an edge-to-edge distance greater than 0.5 cm and were associated with mild perilesional edema. Although spatially close to the previously irradiated parietal bone, they were not in direct contiguity with the calvarial site of stereotactic treatment and were therefore interpreted as new brain metastases (red arrow in Figure 1A).

Accordingly, the three lesions were treated in July 2025 with single-isocenter FSRT to a total dose of 24 Gy in three consecutive fractions of 8 Gy. At the first post-treatment MRI follow-up, all three lesions exhibited marked volumetric enlargement, a confluent appearance, and extensive perilesional edema (red arrow in Figure 1B). In light of this radiological evolution and the overall stability of systemic disease, the patient was referred for neurosurgical evaluation [5]. An ^11^C-methionine positron emission tomography (PET) scan was requested and demonstrated lower tracer uptake compared with that expected in metabolically active metastatic disease, favoring a diagnosis of radiation-induced necrosis rather than tumor progression and helping to clarify the inconclusive MRI findings.

Given the significant symptom burden, manifested by headache, nausea, and diplopia, the patient underwent surgical resection, which resulted in complete resolution of neurological symptoms. Histopathological examination of the surgical specimen definitively confirmed radionecrosis (Figure 1C).

A retrospective review of both FSRT treatment plans revealed that all three subcentimetric brain lesions had developed entirely within the 95% isodose line of the parietal skull metastasis treatment (Figure 1D). This finding was unexpected, particularly considering that a fractionated stereotactic regimen, rather than single-fraction radiosurgery, had been deliberately selected to mitigate the risk of radiation-induced necrosis [6]. Moreover, brain dose constraints had been respected with a substantial safety margin and, to the best of our knowledge, despite theoretical plausibility, cases of brain parenchymal radionecrosis following stereotactic irradiation directed exclusively to skull bone metastases have not been reported to date in the available literature on the topic [7,8,9].

Using an α/β ratio of 3 for brain tissue, the three-lesion cluster developed within a brain region exposed to a biologically effective dose (BED3) of 82.65 Gy during the calvarial metastasis irradiation course, a dose level not typically associated with a high risk of radionecrosis [10]. The subsequent stereotactic treatment delivered a BED3 of 88 Gy.

Moreover, current evidence does not support a clear association between denosumab, cyclin-dependent kinase inhibitors (CDKIs) such as ribociclib, and an increased risk of brain radionecrosis when administered in combination with radiotherapy [1]. The few reported cases of radionecrosis in patients receiving CDKIs involved multiple re-irradiation courses to the same lesions (up to four), suggesting that cumulative radiation exposure and retreatment may represent the primary contributing factors rather than a direct systemic drug–radiation interaction [11].

The timeline of the events in our case is shown in Figure 2.

In conclusion, this unique observation underscores the need for extreme caution when irradiating skull bone metastases, even when using relatively moderate stereotactic doses and fractionated schedules. It highlights a potentially severe adverse effect involving the brain parenchyma that may be easily misinterpreted as intracranial disease progression. Concerns regarding clinically relevant dose spill to the brain during stereotactic irradiation of targets located in immediately overlying tissues therefore appear not only justified, but highly relevant [12].

## Figures and Tables

**Figure 1:**
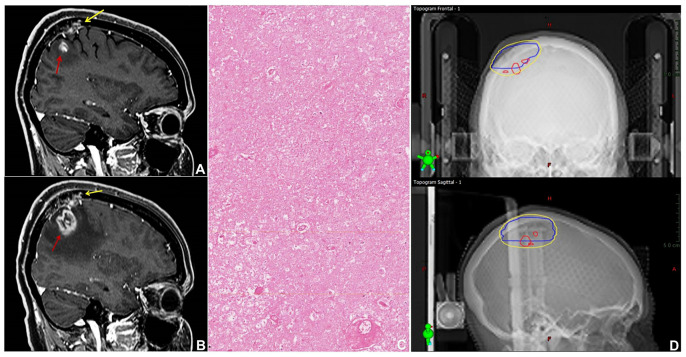
(**A**) MRI from July 2025 showing the outcome of previous radiotherapy on the skull bone metastasis (yellow arrow) and the appearance of three subcentimetric contrast-enhancing lesions within the brain parenchyma. These lesions were not in direct contiguity with the treated skull bone metastasis and were therefore interpreted as brain parenchymal metastases (red arrow indicates one of the three lesions in this sagittal section). (**B**) MRI from October 2025 showing persistent stability of the calvarial lesion after radiotherapy (yellow arrow) and enlargement of the lesion indicated by the red arrow, corresponding to the same lesion in (**A**). (**C**) Histological section showing extensive areas of coagulative necrosis. Hallmark features include marked vascular changes, with wall thickening and hyalinization of small vessels (fibrinoid necrosis), and luminal obliteration in some vessels due to endothelial proliferation or thrombosis. (**D**) Frontal (**top**) and lateral (**bottom**) digitally reconstructed radiographs from the CT simulation, showing the superimposition of the skull bone target (blue), 95% isodose (yellow), and the cluster of three brain parenchymal lesions (red).

**Figure 2:**
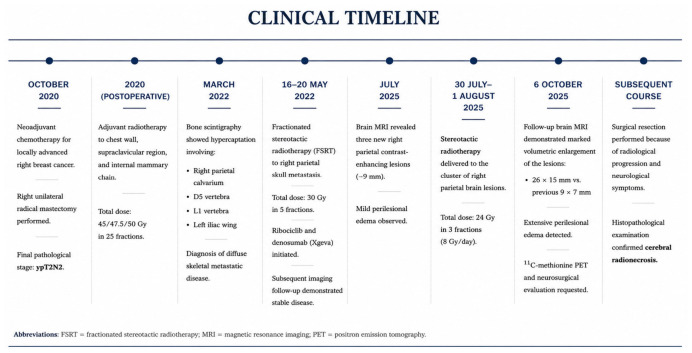
Timeline of the main events in the present case.

## Data Availability

The data that support the findings of this study are available upon request from the corresponding author.

## References

[1] Gagliano A., Prestifilippo A., Cantale O., Ferini G., Fisichella G., Fontana P., Sciacca D., Giuffrida D. (2021). Role of the Combination of Cyclin-Dependent Kinase Inhibitors (CDKI) and Radiotherapy (RT) in the Treatment of Metastatic Breast Cancer (MBC): Advantages and Risks in Clinical Practice. Front. Oncol..

[2] Zagardo V., La Fauci D., Umana G.E., Lavalle S., Palmisciano P., Noto M., Boncoraglio A., Scalia G., Ferini G. (2025). Dosimetric Comparison of Coplanar, Non-coplanar, and Mixed-Arc VMAT for Head and Face Skin Cancers: A Multi-scenario Analysis. Cancer Diagn. Progn..

[3] Milano M.T., Grimm J., Niemierko A., Soltys S.G., Moiseenko V., Redmond K.J., Yorke E., Sahgal A., Xue J., Mahadevan A. (2021). Single- and Multifraction Stereotactic Radiosurgery Dose/Volume Tolerances of the Brain. Int. J. Radiat. Oncol. Biol. Phys..

[4] Faruqi S., Ruschin M., Soliman H., Myrehaug S., Zeng K.L., Husain Z., Atenafu E., Tseng C.-L., Das S., Perry J. (2020). Adverse Radiation Effect After Hypofractionated Stereotactic Radiosurgery in 5 Daily Fractions for Surgical Cavities and Intact Brain Metastases. Int. J. Radiat. Oncol. Biol. Phys..

[5] Umana G.E., Ranganathan S., Marrone S., Naimo J., Giunta M., Spitaleri A., Fricia M., Ferini G., Scalia G. (2025). Optimizing Strategies in Patients Affected by Tumors Infiltrating the Skull: A Single Center Experience. Brain Sci..

[6] Inserra F., Barone F., Palmisciano P., Scalia G., Da Ros V., Abdelsalam A., Crea A., Sabini M.G., Tomasi S.O., Ferini G. (2022). Hypofractionated Gamma Knife Radiosurgery: Institutional Experience on Benign and Malignant Intracranial Tumors. Anticancer Res..

[7] Kotecha R., Angelov L., Barnett G.H., Reddy C.A., Suh J.H., Murphy E.S., Neyman G., Chao S.T. (2014). Calvarial and skull base metastases: Expanding the clinical utility of Gamma Knife surgery. J. Neurosurg..

[8] Scalia G., Porzio M., Costanzo R., Giurato E., Gibilisco F., Iacopino D.G., Maugeri R., Nicoletti G.F., Umana G.E., Alessandrello R. (2025). Large Skull Metastasis in Follicular Thyroid Carcinoma: A Comprehensive Case Presentation and Systematic Review. J. Neurol. Surg. A Cent. Eur. Neurosurg..

[9] Park D.J., Voruganti H., Annagiri S., Shaghaghian E., Hori Y.S., Persad A.R., Yoo K.H., Abu-Reesh D., Lam F.C., Tayag A. (2025). Efficacy and Safety of CyberKnife Stereotactic Radiosurgery for Occipital Condyle Metastasis. Neurosurg. Pract..

[10] Palička M., de Jong A.M., David S., Rybář M., Jackaninová J., Knybel L., Reguli Š., Blažek T., Tomoszková S., Verhoeff J.J.C. (2026). Incidence of radiation necrosis following different radiotherapy fractionation schedules for intracranial meningiomas. Strahlenther. Onkol..

[11] Figura N.B., Potluri T.K., Mohammadi H., Oliver D.E., Arrington J.A., Robinson T.J., Etame A.B., Tran N.D., Liu J.K., Soliman H. (2019). CDK 4/6 inhibitors and stereotactic radiation in the management of hormone receptor positive breast cancer brain metastases. J. Neurooncol..

[12] Ferini G., Fichera C., Boncoraglio A., Umana G.E., Forte S. (2024). De Felice scheme: No risk at all of brain radionecrosis?. Head Neck.

